# The Use of Zirconia for Implant-Supported Fixed Complete Dental Prostheses: A Narrative Review

**DOI:** 10.3390/dj11060144

**Published:** 2023-06-01

**Authors:** Chiara Cinquini, Fortunato Alfonsi, Vincenzo Marchio, Francesco Gallo, Francesco Zingari, Alessandro Remigio Bolzoni, Stefano Romeggio, Antonio Barone

**Affiliations:** 1 Department of Surgical, Medical and Molecular Pathology and Critical Care Medicine, University of Pisa, 56126 Pisa, Italy; 2 Department of Maxillofacial Surgery, Istituto Stomatologico Italiano, 20122 Milan, Italy; 3 Department of Maxillofacial Surgery, Galeazzi Hospital, 20157 Milan, Italy; 4 Maxillo-Facial Surgery and Dental Unit, Department of Biomedical, Surgical and Dental Sciences, University of Milano, 20122 Milan, Italy; 5 Fondazione IRCCS Ca’ Granda Ospedale Maggiore Policlinico di Milano, University of Milano, 20122 Milan, Italy; 6 Private Practice, Innova Clinique, 28845 Domodossola, Italy

**Keywords:** zirconia, full-arch rehabilitation, dental implants, prosthesis

## Abstract

The success of implant-supported fixed complete dental prostheses (ISFCDPs) depends on multiple factors: some are related to the fixtures, such as fixture material, surface characteristics, positioning, and type of connection to prosthetic components; others are related to the prostheses, such as design and materials used. Zirconia is a material widely used in fixed prosthodontics, whether on natural teeth or on implants, with excellent results over time. Regarding the use of zirconia for ISFCDPs, the 2018 ITI Consensus Report stated that “implant-supported monolithic zirconia prostheses may be a future option with more supporting evidence”. Since CAD/CAM technology and zirconia are being continuously innovated to achieve better results and performances over time, a narrative review of the literature seems necessary to focus research efforts towards effective and durable solutions for implant-supported, full-arch rehabilitations. The objective of the present narrative review was to search the literature for studies regarding the clinical performance of zirconia-based ISFCDPs. According to the results of this review, the use of zirconia for ISFCDPs showed good clinical outcomes, with high survival rates ranging from 88% to 100% and prosthetic complications that were restorable by the clinicians in most cases.

## 1. Introduction

The increasing aging of the population worldwide has led to a major proportion of patients who could develop complete edentulism and consequently need prosthetic rehabilitation, which can be an actual challenge for clinicians [1,2].

Before the advent of implant dentistry, the only therapeutic option to treat complete edentulism was to manufacture complete removable dentures [3].

Despite many patients still accepting complete dentures for financial or personal reasons, some physiological modifications of the normal anatomy of the maxillary and mandibular bones, such as severe alveolar bone resorption, could lead to difficulties in prosthetic retention, which in turn causes pain and difficulties in chewing and eating properly [4].

Nowadays, thanks to the high survival and success rates of dental implants [5], implant-supported fixed complete dental prostheses (ISFCDPs) or implant-retained removable dentures have become a valid alternative to complete removable dentures to overcome the above-mentioned issues [6].

The advantages offered by ISFCDPs are multiple, ranging from the improvement of patients’ comfort to the preservation of the alveolar bone from the mechanical stress of removable dentures, among others [6,7].

For this reason, according to some authors [8], ISFCDPs should be considered the gold standard of care, especially in clinical scenarios characterized by severe atrophy of the alveolar bone. In implant prosthodontics, the result of a fixed full-arch rehabilitation depends on multiple factors: some are related to the fixtures, such as fixture material, surface characteristics, positioning, and type of connection to prosthetic components; others are related to the prostheses, such as design and materials used.

Some of the most commonly used materials for ISFCDPs are metal-acrylic hybrids, where acrylic resin teeth and gingival tissue are cemented on a metal framework; these materials are easy to use, affordable, and can be easily repaired [9]. On the other hand, they are characterized by relatively high rates of complications such as teeth debonding from the structure, fractures, and screw/abutment loosening [10].

Zirconia is a material widely used for fixed prostheses supported by natural teeth or implants, with excellent results over time [11]. Moreover, zirconia showed good biocompatibility and high flexural strength, together with less accumulation of dental plaque and resistance to staining compared to resinous materials [12].

According to the Group 2 ITI Consensus Report for Prosthodontics and Implant Dentistry of May 2018 [13], zirconia can be used as either a fixture material or a prosthetic material. One-piece zirconia implants are recommended only in certain clinical conditions, while there are no sufficient data regarding two-piece zirconia implants to recommend their clinical use without caution. The performance of zirconia used as a prosthetic material for implant-supported single crowns has, instead, been deemed similar to that of metal-ceramic crowns; it has been noted, however, that a higher percentage of complications arise with zirconia-ceramic crowns compared to metal-ceramic crowns and monolithic zirconia crowns.

Regarding the use of zirconia for full-arch rehabilitations, the 2018 ITI Consensus Report states that “implant-supported monolithic zirconia prostheses may be a future option with more supporting evidence”.

In a more recent review [14], the author confirms that implant-supported monolithic zirconia full-arch restorations may solve the ceramic chipping issue of zirconia-ceramic and metal-ceramic prostheses, especially if certain design requirements are respected: Multiple studies with long-term follow-up have been published, and the results are promising.

Since CAD/CAM technology and zirconia are being continuously innovated to achieve better results and performance over time, a narrative review of the literature seems necessary to focus research efforts towards effective and durable solutions for implant-supported, full-arch rehabilitations.

The objective of the present narrative review was to search the literature for clinical studies focusing on the performance in terms of survival, success, and prosthetic complications of zirconia-based implant-supported fixed complete dental prostheses.

## 2. Materials and Methods

The research was conducted by two groups of reviewers (Group 1: CC/VM/FG; Group 2: AB/FA/SR). The two groups of reviewers attended a group meeting to choose the PICO question, to be aligned during the article selection, and to set up the inclusion/exclusion criteria.

The selected PICO question was the following: “What are the clinical performances in terms of survival, success and prosthetic complications of zirconia-based ISFCDPs?”.

The following inclusion/exclusion criteria were set up:

Inclusion criteria:

- Human clinical studies (prospective studies, retrospective studies, randomized clinical trials, and case series);
- Articles evaluating the clinical outcomes of zirconia-based implant-supported full-arch rehabilitations or comparing zirconia to other restorative materials;
- Articles published in English.
- Exclusion criteria:
- Animal studies;
- Case reports;
- Articles published in languages other than English.

The two groups of reviewers conducted a double Medline search through PubMed on literature published up to December 2022. No starting year was chosen in order to include as many results as possible.

Group 1 (CC/VM/FG) conducted the research using the following keywords: “prosthetics, dentistry, zirconia, implant supported NOT zirconia implants”, obtaining 220 results.

Group 2 (AB/FA/SR) conducted the research using the keywords “implant, full arch rehabilitation, zirconia”, obtaining 30 results.

In the two groups, each member performed the search by themselves and then compared it with other members of the same group, deleting duplicates.

Then, the results of both research groups were compared, and the duplicates were eliminated.

Finally, a total of 248 articles were selected for title screening.

**Figure 1:**
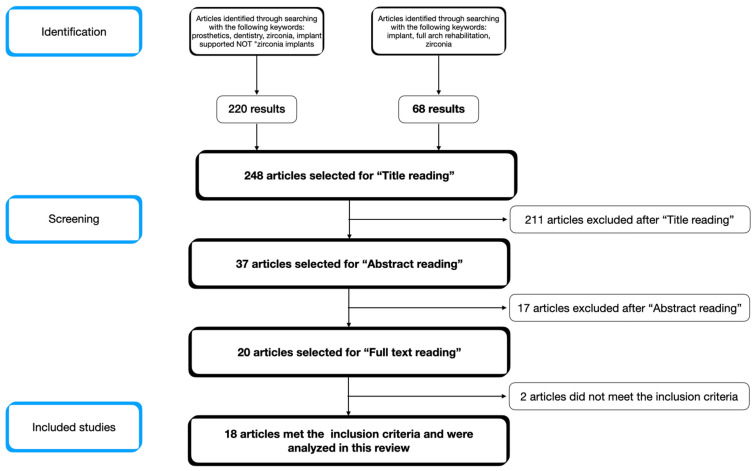
Flowchart of the search strategy and selection process for the articles.

After the title screening, performed by both groups of reviewers, the results were compared and discussed. Finally, 37 articles were selected for abstract reading. After the abstract reading, 17 articles were excluded, and 20 articles were considered eligible for full-text reading. Following the full-text reading, the results were discussed among the reviewers. Two articles did not meet the inclusion criteria, and 18 articles were finally included in this review. Figure 1 shows the flowchart of the search strategy and selection of the articles included in this review.

## 3. Results

All the included articles were published between 2010 and 2021.

The main results of the studies included in this review are visible in Table 1.

The first study included, by Larsson C. et al. [15], was a prospective study conducted on 10 patients requiring mandibular full-arch rehabilitation. The authors evaluated the clinical outcomes of prosthetic rehabilitations made of yttria-stabilized tetragonal zirconia polycrystal (Y-TZP), a material possessing the characteristic of inhibiting the propagation of micro-cracks thanks to its tetragonal phase. After three years of follow-up, all the rehabilitations were in use and the patients were satisfied; however, superficial chipping of the ceramic was observed in 9 out of 10 patients (90%). The authors concluded that this type of restoration should be carefully proposed, and studies with longer follow-up are required to better understand the factors involved in the chipping of the structures.

Oliva J. et al. [16] reported the retrospective analysis with 5 years of follow-up of zirconia full-arch rehabilitations over three implants positioned in the mandible or maxilla (all-on-three protocol). They analyzed 24 full-arch rehabilitations placed with a delayed loading protocol in 17 patients. Except for two patients, all the cases were restored 3 months after surgery with full-zirconia frameworks with a ceramic veneer on the buccal aspect for esthetic reasons. After 5 years of function, the authors found a survival rate of 100% and a success rate of 100%, reporting no fractures of the frameworks and only two minor prosthetic complications (screw loosening in one case and chipping of the ceramic layer in another one); however, all the patients wore a nightguard to prevent fractures or chipping.

In the third included article by Papaspyridakos P. and Lal K. [17], the authors reported the clinical outcomes of CAD/CAM-manufactured zirconia full-arch rehabilitations after 2–4 years (mean follow-up of 36 months) of follow-up. The authors included 14 patients who had been rehabilitated with dental implants and CAD/AM porcelain fused to zirconia ISFCDPs in the mandible and/or maxilla between 2007 and 2009.

The authors observed a 100% survival rate up to 4-year follow-up and a ceramic chipping rate of 31.25%, mostly minor (3 out of 4 patients) and requiring only polishing or restoration with composite resin. Only one case of porcelain fracture required laboratory repair. The authors identified three risk factors associated with porcelain chipping: patients’ parafunctional activities, the absence of a nightguard, and the presence of another ISFCDP as an antagonist.

Pozzi and colleagues [18] reported the results of a retrospective study conducted on 22 patients who were rehabilitated with 26 CAD/CAM ISFCDPs made of zirconia with ceramic veneering. After 36 to 60 months of follow-up (mean follow-up = 42.3 months), the authors reported a survival rate of both implants and prostheses of 100% and a cumulative prosthetic success of 89%, due to ceramic chipping occurring in 3 cases (11%). No prosthesis had to be replaced or repaired at the laboratory, and all the patients were satisfied with the functional and aesthetic outcomes of their rehabilitations.

In the fifth included article, Limmer and colleagues [12] conducted a prospective study on 17 edentulous patients requiring both maxillary and mandibular prostheses. The treatment plan was a total upper denture and an ISFCDP at the mandibular level. The patients were finally rehabilitated with an implant-supported monolithic zirconia fixed dental prosthesis (MZ-FDP) at the mandibular level and a removable denture in the maxilla. At 1 year of follow-up, the implant survival rate was 99% at implant level (only one implant failed), and prosthetic complications occurred in 10 out of 17 patients (58.8%), mostly tooth chipping of the opposing removable prosthesis, abutment loosening, fracture of the abutments, and debonding of the prosthetic components. Only one MZ-FDP fractured six months following the insertion, and another one was lost due to implant failure.

In another retrospective clinical study [19] conducted on 95 patients requiring zirconia-based implant-supported fixed rehabilitations (both fixed partial dentures, single crowns, and full arch rehabilitations), the authors evaluated the complications rate of 156 implant-supported prostheses; among them, 11 were maxillary full-arch rehabilitations. All the prostheses were made with CAD/CAM processes to obtain a zirconia framework, and a full veneering with felspathic ceramic was placed. Only one case of extensive chipping of the veneering material was observed in one bruxing patient (9%), requiring the fabrication of a new prosthesis made of titanium with acrylic veneering to prevent chipping problems. No extensive framework fractures were observed among this cohort of rehabilitations (11), leading to a survival rate of 91%.

Venezia P. and colleagues [20] performed a retrospective analysis of 26 implant-supported full-arch zirconia prostheses with ceramic veneering limited to the buccal aspect. Eighteen patients treated between 2010 and 2013 were re-evaluated after 10 to 36 months, with a mean follow-up of 20.9 months. Eleven prostheses were designed with ceramic veneered incisal margins on the anterior teeth, while the remaining 15 had zirconia incisal margins. No implant was lost at follow-up, and the overall implant success rate was 94.8%. Minor ceramic chipping was observed in three cases (11.5%), thus not affecting the aesthetic and functional results and requiring only low-speed polishing. No fracture of the zirconia framework was observed, leading to a prosthesis survival rate of 100%.

In the retrospective study by Tartaglia G.M. and colleagues [21], the authors re-evaluated 113 patients who received one or two implant-supported immediately loaded full-arch prostheses made of resin or zirconia. Two hundred fourteen protheses were evaluated after five years of follow-up. All the patients had been rehabilitated with 4 to 6 maxillary/mandibular implants and an implant-supported rehabilitation made of a polymethyl methacrylate (PMMA) framework veneered with resin or a full zirconia framework veneered with ceramic. All the patients wore a provisional acrylic screw-retained prosthesis and, after four months, received the final restoration. The selection between the two types of prosthesis was made based on the occurrence of the facture of the provisional prosthesis (16 patients) or the high patients’ esthetic expectations (32 patients); therefore, 48 patients received the zirconia prosthesis. At five years of follow-up, the authors observed a complication-free survival rate of 60.5% for the zirconia prosthesis and of 78.1% for the PMMA prosthesis. The overall survival rates were 84.7% and 88.9%, respectively, for PMMA and zirconia prostheses. The authors concluded that both materials were clinically successful at 5-year follow-up.

In the retrospective study performed by Rojas Vizcaya F. [22], 10 patients each receiving two monolithic full-arch zirconia prostheses were evaluated. The patients were divided into two groups: one in which the prostheses did not have any cut-back for veneering ceramic (10 prostheses) and one in which nonfunctional cut-back, meaning that the cut-back in the monolithic zirconia excluded incisal margins and occlusal surfaces, was executed to apply veneering ceramic. The follow-up was from a minimum of 2 to a maximum of 7 years. A total of 100% prosthetic and implant survival and success were reported; however, one prosthetic complication was reported for each group: gingival pink ceramic chipping in the non-cutback group and screw loosening in the cutback group.

Papaspyridakos P. et al. [23] designed a prospective study to examine the performance over two years of five full arch rehabilitations in a total of three patients. The prostheses were monolithic zirconia with nonfunctional cutback supported by six implants per arch and were digitally designed and separated into three sections (two posterior, one frontal). Over the 2-year period of follow-up, only one case of minor porcelain chipping was reported, and it was repaired by polishing the margins.

In another retrospective study, Gonzalez J. and Triplett R. [24] included 40 patients treated with a total of 56 full arch prostheses supported by implants, 44 of which were made from nonfunctional cutback monolithic zirconia with ceramic veneers and 12 with conventional metal-acrylic cores and acrylic crowns. The mean follow-up was 2.75 years. The patients were divided into groups based on which materials were in contact during occlusion: maxillary zirconia vs. mandibular zirconia (4 patients, 8 prostheses); maxillary zirconia vs. mandibular natural teeth (24 patients, 24 prostheses); maxillary zirconia vs. mandibular metal-acrylic (12 patients, 24 prostheses). No survival nor success rates were clearly expressed, but prosthetic complications were reported: for the zirconia vs. zirconia group, there was one minor porcelain chip and one debonded metal insert. For zirconia vs. natural dentition, there were 5 minor porcelain chippings and 1 debonded metal insert in six out of 24 prostheses. For the zirconia vs. metal-acrylic group, only complications on metal-acrylic arches were reported: 16 tooth fractures among 12 prostheses. Overall, the zirconia prostheses showed 6 minor porcelain fractures and 2 debonded inserts, while for the metal acrylic arches, 16 tooth fractures were reported. The authors concluded that monolithic zirconia for implant-supported full arch prostheses is viable, but particular attention should be given to the opposing arch: using different materials could increase prosthetic complications over time.

Box V. et al. [25] published a retrospective study in 2018 in which 37 patients, treated with a total of 49 full-arch implant-supported prostheses, were evaluated with a follow-up of a minimum of 1 to a maximum of 5.8 years. Four groups are reported: 22 metal acrylic arches, 14 “retrievable crown” arches (full coverage restorations cemented on milled zirconia or titanium bars), 7 true monolithic zirconia arches, and 6 nonfunctional cutback porcelain veneered zirconia arches. A total of 6 failures (meaning that the prostheses need to be made again and are not repairable) were reported, 2 for each group except for the true monolithic zirconia group. Regarding the prosthetic complications, they were distributed as follows: 12 complications in 22 prostheses in the metal-acrylic group, 10 complications out of 14 prostheses in the retrievable crown group, 2 complications out of 7 prostheses in the true monolithic zirconia group, and 5 complications out of 6 prostheses in the nonfunctional cutback zirconia group. The most common complications for each group were: for metal acrylic prostheses, posterior tooth wear; for true monolithic zirconia wearing of opposing restorations; for retrievable crown prostheses, chipping, fracturing, and debonding of crowns; for nonfunctional cutback porcelain veneered zirconia, chipping of opposing restorations. The authors concluded that monolithic zirconia had the lowest incidence of complications and that attention should be given to the type of material selected for a full-arch, implant-supported restoration. It is important to note, however, that it was unclear whether the retrievable crown prostheses had a titanium or zirconia bar.

In a prospective study, Caramês J. et al. [26] recruited 150 patients, who were rehabilitated with 193 implant-supported full-arch prostheses. They were divided into two groups: 83 prostheses were made with monolithic zirconia fully veneered with ceramic, while the remaining 110 were made with nonfunctional cutback monolithic zirconia veneered with ceramic. The mean follow-up was 1.6 ± 0.5 years for the ceramic veneered zirconia prostheses and 1.5 ± 0.5 years for the nonfunctional cutback zirconia prostheses. The success rate was over 99% for both groups, and no statistically significant difference in terms of complications or performance was reported. A total of 20 prosthetic complications were reported, 10 for each group, for a total complication rate of 11.3%. The authors also highlighted that 85% of these complications occurred when the opposing arch was also an implant-supported rehabilitation.

Barootchi et al. [1], in a retrospective study from 2020, collected data from 74 full-arch, implant-supported prostheses supported by six or more implants. Two groups were identified: 43 metal acrylic prostheses and 31 zirconia prostheses of the “retrievable crown” design (monolithic zirconia framework with full coverage cemented ceramic crowns). The mean follow-up was 8.7 ± 3.37 years, with a minimum follow up of 5 years. The survival rates at 5 years for the zirconia prostheses were 93.7 ± 5.5%, while at 8 years they were 88 ± 8.8%. For the metal acrylic prostheses, survival rates at 5 years were 83 ± 11.1%, and at 8 years they were 67.6 ± 14.8%. Regardless of the material, maxillary arches showed a higher survival rate as compared to mandibular arches. The authors reported a statistically significant difference in the occurrence of minor complications, which were more frequent in metal-acrylic prostheses than in zirconia prostheses (72.1% vs. 61.3%); the same was observed for major prosthetic complications, which needed lab work to be repaired (41.9% in metal-acrylic vs. 25.8% in zirconia). After adjusting for the different follow-up times, which were longer for metal-acrylic prostheses, no statistically significant difference was found between the two materials.

Another retrospective study from Capparè P. and colleagues [27], in 2020, included 50 patients with 50 full-arch, implant-supported prostheses (22 maxillary, 28 mandibular) and divided them into 2 groups according to the material used: 25 were made with nonfunctional cutback monolithic zirconia veneered with ceramic; the other 25 were metal acrylic. The minimum follow-up was 2 years, and neither complications nor failures were reported. The authors concluded that nonfunctional cutback monolithic zirconia was comparable to metal-acrylic for the realization of full-arch implant-supported prostheses.

Diéguez-Pereira M. et al. [28], in 2020, designed a retrospective study that included multiple types of fixed zirconia restorations. Forty-eight patients with a total of 58 full-arch implant-supported prostheses were included. Two groups were identified: 14 prostheses made of true monolithic zirconia and 44 made of ceramic-veneered monolithic zirconia. A success rate of 81.3% was reported, and only 1 case of minor prosthetic complications (chipping) was reported. No comparison between the two designs was made. The authors concluded that monolithic or partially veneered zirconia both have good clinical behavior, but more clinical studies are needed to confirm their findings.

Pozzi A. et al. [29] published a retrospective study in 2021 in which 98 patients received a total of 111 full-arch implant-supported prostheses. Of these, 24 were made with a monolithic zirconia framework veneered with ceramic, 72 were partial implant-supported bridges, and 15 were full arches with titanium bars. The mean follow-up time was 7.2 ± 3.4 years. The cumulative prosthetic success rate was 91.9%, and no zirconia fractures were reported. The authors concluded that zirconia-based screw-retained implant-supported prostheses are a reliable long-term treatment option for partial and complete edentulism.

Tirone F. et al. [30], in 2021, designed a retrospective study including 140 patients and 180 implant-supported full arch prostheses. Three groups were identified: group 1, with 21 completely veneered zirconia framework prostheses; group 2, with 71 cutback monolithic zirconia with ceramic veneering; and group 3, with 41 true monolithic zirconia with ceramic veneering only in the gingival portion. The mean follow-up time was 3.4 ± 1.7 years, and a 93.3% success rate was reported. A total of 10 prosthetic failures were reported, 2 caused by implant failures and 8 by framework fractures. The authors concluded that, to make zirconia a viable material for full-arch implant-supported restorations, some dimensional parameters need to be followed. The ratios between cantilever length and cross-sectional connector area should be less than 0.51, while the ratio between the cantilever length and screw access opening length should be less than 1.48.

## 4. Discussion

Porcelain-fused-to-metal (PFM) has been used for years for implant-supported rehabilitations and can still be considered the “gold standard” for this kind of restoration due to its history of excellent results and performance over time [19,31]. In recent years, however, the use of zirconia as a framework for full-arch rehabilitations has become very popular due to zirconia’s good mechanical properties and the possibility of fabrication with a digital workflow, since CAD/CAM fabrication of monolithic zirconia structures is widely available [21]. According to recent literature, zirconia can be used mainly in two solutions:

- Monolithic form, where no additional ceramic is added and esthetics are provided by the zirconia itself and glazes applied by the technician;
- Veneered with highly esthetic glass ceramic by creating a cutback in the monolithic structure, either functional (veneering of buccal, occlusal, and marginal surfaces) or non-functional (veneering of the buccal surface) [23,32].

Other solutions include milling a monolithic zirconia framework on which full coverage glass ceramic crowns are then luted.

Unfortunately, zirconia is not exempt from mechanical complications such as chipping or delamination of the ceramic veneering, if present, and fracture of the framework if not properly designed [33].

According to the Group 2 ITI Consensus Report for Prosthodontics and Implant Dentistry (2018) [13], zirconia can be used as either a fixture material or a prosthetic material. One-piece zirconia implants are recommended only in certain clinical conditions, while there is not sufficient data regarding two-piece zirconia implants to recommend their clinical use without caution. The performance of zirconia used as a prosthetic material for implant-supported single crowns has, instead, been deemed similar to that of metal-ceramic crowns; a higher percentage of complications was noted with zirconia-ceramic crowns compared to metal-ceramic crowns and monolithic zirconia crowns.

At the present time, a growing number of studies reporting the use of zirconia in ISFCDPs can be found in the literature, but the number of treated patients is still scarce, and the follow-up times are often short [34].

In addition, most if not all the studies included in this review do not clearly explain which kind of zirconia is used. Since 3YTZP, 5YTZP, and other types of zirconia have different esthetic and mechanical properties, the absence of a specification on which type of zirconia has been used for the restoration in the selected manuscripts should be considered a source of bias.

One of the most common complications of full-zirconia rehabilitations with ceramic veneering is the chipping of the ceramic layer [35].

In 2018, the ITI Consensus Report stated that “implant-supported monolithic zirconia prostheses may be a future option with more supporting evidence” [13]. In a recent literature review [14], the author confirms that implant-supported, monolithic zirconia full-arch rehabilitations may solve the ceramic chipping issue of zirconia-ceramic and metal-ceramic prostheses, especially if certain design requirements are respected: Multiple 5-year follow-up studies have been published, and the results are promising.

According to the articles included in this review, the survival rate of full-arch zirconia rehabilitations (intended as the presence of the prosthesis in the oral cavity with no need for replacement) varies from 88% to 100% with a follow-up period ranging from 1 to 7 years.

Regarding the success rates, all the studies included in this review showed great heterogeneity in the definition and subsequent reporting of restorations’ success rates. In addition, some articles did not specify the definition of prosthetic success rates. Some authors defined prosthetic success as the total absence of prosthetic complications (even minor complications such as screw loosening or ceramic chipping requiring only polishing), while others considered the cases prosthetic successes even if some minor complications occurred. We reported, if present, the percentages of prosthetic success expressed by the authors in their work; however, in many cases, they cannot be compared.

The heterogeneity in prosthetic designs for full-arch fixed restorations made of monolithic zirconia is highlighted by the results of the present narrative review: The plethora of designs, such as completely monolithic zirconia, functional or non-functional cutback veneered zirconia, retrievable crown prostheses with a zirconia framework, etc., hinder the capability of researchers to properly evaluate the performance of such rehabilitations.

We believe that a unique definition of ISFCDPs’ success should be evaluated, considering that a restoration requiring no replacement or laboratory repair and fulfilling patients’ expectations in terms of esthetic and functional aspects should be considered successful. This needs to be carried out by presenting a clear and repeatable procedure and comparing different factors that were not considered in many of the included studies, such as patient type (high or low masticatory forces), parafunction (bruxism), opposing arch materials, number and orientation of supporting implant fixtures, and methods of design and production.

## 5. Conclusions

According to this narrative review of the literature, the use of zirconia for ISFCDPs showed good clinical results with high survival rates (88–100%). The major complication (superficial chipping) was, in most cases, easily restorable directly by the clinicians, with no necessity for laboratory repair. It is worth noting, however, that the authors found no randomized clinical trials comparing the clinical performances of different types of ISFCDPs made of zirconia or comparing zirconia to different materials. Most of the studies included in the review, however, have a small number of cases; thus, the evidence in this review might be influenced by biases found in the selected studies and should be accepted with caution. To design an adequate randomized clinical trial, a repeatable and clear procedure for the rehabilitation project, implant placement, and prosthetic design should be introduced.

In conclusion, more research needs to be conducted regarding zirconia-based ISFCDPs to assess whether this material can be used in all cases or only in specific clinical situations.

## Figures and Tables

**Table 1:** Characteristics of the included studies.

Reference	Type of Study	Number of Patients	Number and Type of ISFCDPs	Experimental Groups/Type of Restorative Material Used	Follow-Up	Prosthetic Survival Rate	Prosthetic Success Rate	Prosthetic Complications
Larsson C. et al. (2010) [15]	Prospective Clinical Study	10 patients	10 mandibular arches	Yttria-stabilized tetragonal zirconia polycrystal (Y-TZP) ISFCDPs (Cercon technique)	3 years	100%	66%	Superficial chipping (90%); marginal integrity was considered excellent in 70% of the cases and acceptable in 30%. No prosthesis was lost, fractured, or required laboratory repair.
Oliva J. et al. (2012) [16]	Retrospective Clinical Study	17 patients	24 arches	Full zirconia framework + ceramic on the buccal aspect	5 years	100%	100%	Buccal ceramic chipping (4.1%) Screw loosening (4.1%)
Papaspyridakos P. and Lal K. (2013) [17]	Retrospective Clinical Study	14 patients	16 arches (10 in mandible, 6 in maxilla)	Porcelain fused to zirconia (PFZ)	2–4 years	100%	Not specified	Ceramic chipping (31.25%)
Pozzi et al. (2015) [18]	Retrospective Clinical Study	22 patients	26 arches (14 in mandible, 12 in maxilla)	Full Zirconia framework + ceramic veneering	>3 years (36 to 60 months)	100%	89%	Ceramic chipping (11%)
Limmer B. et al. (2014) [12]	Prospective Clinical Study	17 patients	17 mandibular arches	Monolithic zirconia fixed dental prosthesis (MZ-FDP)	1 year	88%	Not specified	Prosthetic complications (58.8%), including tooth chipping of the opposing removable denture, abutment loosening, fracture of the abutments, and debonding of the prosthetic components. One prosthesis was lost due to fracture and one due to implant failure (12%).
Worni A. et al. (2015) [19]	Retrospective Clinical Study	90 patients	156 screw-retained zirconia prosthesis (11 maxillary full-arches)	Full Zirconia framework + ceramic veneering	2–7 years	91%	Not specified	Extensive ceramic chipping in one maxillary arch (9%). No framework fracture was observed.
Venezia P. at al. (2015) [20]	Retrospective Clinical Study	18 patients	26 arches (17 in maxilla and 9 in mandible)	Full zirconia framework + ceramic on the buccal aspect	10 to 36 months (mean = 20.9 months/1.6 years)	100%	Not specified	Minor ceramic chipping (11.5%)
Tartaglia G.M. et al. (2016) [21]	Retrospective Clinical Study	113 patients	214 arches (105 maxillary, 109 mandibular)	Group 1: polymethyl methacrylate (PMMA framework) veneered with resin (166 prostheses on 96 patients) Group 2: Full Zirconia framework + ceramic veneering (48 prostheses on 32 patients)	5 years	88.9%	60.5%	37 out of 113 patients (32.7%) had prosthetic complications (reparable fractures, abutment-prosthesis screws loosening, ceramic chipping for zirconia prostheses); the prosthetic material did not influence the risk of developing complications.
Rojas Vizcaya F. et al. (2018) [22]	Retrospective Clinical Study	10 patients	20 arches	Monolithic full arch zirconia prostheses10 with partial cutback10 without cutback	2–7 years	100% implant and prosthetic	100%	one prosthetic complication was reported for each group: gingival pink ceramic chipping in the non-cutback group and screw loosening in the cutback group.
Papaspyridakos P. et al. (2018) [23]	Prospective Clinical Study	3 patients	5 arches	Monolithic zirconia with mild facial porcelain veneering, not full arch but multiple FDPS	2 years	Implant and prosthetic 100%	100%	1 porcelain chipping
Gonzales J. and Triplett R. (2017) [24]	Retrospective Clinical Study	40 patients	56 arches 44 zirconia and 12 hybrid prostheses (metal-acrylic)	Maxillary ZIRCAP and mandibular ZIRCAP (4 patients, 8 prostheses)Maxillary ZIRCAP and mandibular natural dentition (24 patients) Maxillary ZIRCAP and mandibular conventional hybrid prosthesis (metal-acrylic) (12 patients)	Mean of 2.75 years (33 months)	100% (extrapolated)	100% (extrapolated)	MZIRCAP vs. MZIRCAP 1 minor porcelain chipping and 1 debonded metal insert MZIRCAP vs. NATDENT 6/24 complications, 5 minor porcelain chippings and 1 debonded metal insert MZIRCAP vs. metal-acrylic no complications, but complications on metal acrylic: 16 tooth fractures among 12 prostheses. ZIRCAP 6 minor porcelain fractures and 2 debonded inserts, metal acrylic 16 tooth fractures.
Box V. et al. (2018) [25]	retrospective	37 patients	49 arches	22 metal acrylic 14 retrievable crown 7 monolithic zirconia 6 porcelain veneered zirconia	Between 1 and 5.8 years (12 to 70 months)	Not specified	Not specified	12/22 for metal acrylic, 10/14 for retrievable crown, 2/7 monolithic zirconia, 5/6 porcelain veneered zirconia Fractured teeth highest in retrievable crown (6/14) then metal-acrylic (4) then PVZ (3). The most common complications were: (1) MA: posterior tooth wear, highest in metal acrylic (10), then retrievable crown (3), then PVZ (2), then monolithic zirconia (1) (2) RC: chipping and fracturing of the restorations, debonding of crowns (4) (3) MZ: wear of opposing restorations, and (4) PVZ: chipping of opposing restorations.
Caramês J. et al. (2019) [26]	Prospective Clinical Study	150 patients	193 arches	83 ceramic-veneered zirconia full arches (PVZ) 110 buccal-veneered (nonfunctional) monolithic zirconia full arches (MZ)	608.80 ± 172.52 days for PVZ (1.66 ± 0.47 yrs) 552.63 ± 197.57 days for MZ (1.51 ± 0.5 days)	Over 99% for both groups.	Not specified	PVZ group: 10 MZ group: 10 Total complication rate: 11.3%. Most common: -loss of access chamber composite plug -screw loosening 85% of the complications occurred when the opposing arch consisted also of a full-arch implant-supported rehabilitation.
Barootchi S. et al. (2020) [1]	Retrospective Clinical Study	56 patients	74 full arches	43 metal-acrylic 31 zirconia	Min 5 years (mean 8.7 ± 3.37)	AT 5 YEARS: Zirconia: 93.7 ± 5.5% Metal acrylic: 83.0 ± 11.1% AT 8 YEARS Zirconia: 88 ± 8.8% Metal acrylic: 67.6 ± 14.8%		Metal acrylic: 94 single tooth fracture/dislodgement in 22 prostheses. Zirconia: single tooth chipping fracture (36 times in 9 prostheses). Minor complications 67.6%, major complications 35.1%. Major complications: multiple teeth fracture requiring lab work (40 times in 17 metal-acrylic prostheses, 17 times in 4 zirconia fixed prostheses) More minor complications in metal-acrylic than zirconia (72.1% vs. 61.3% P = 0.329) mean of 3.4 vs. 1.7 minor complications PER CASE. Major complications more common in metal-acrylic prostheses than in zirconia ones (41.9% vs. 25.8%) No statistical significance after adjusting for the different follow-up times.
Capparè P. et al. (2021) [27]	Retrospective Clinical Study	50 patients	50 arches (22 maxillary, 28 mandibular)	25 Monolithic zirconia with ceramic veneering limited to non-functional areas. 25 metal-acrylic	Minimum 2 years (average not specified)	100%	100%	No complications reported
Diéguez-Pereira et al. (2020) [28]	Retrospective Clinical Study	48 patients (it is not specified how many have full arch rehabilitations)	58 arches (14 monolithic 44 partially veneered) 154 restorations were included in the study. (82 monolithic and 72 with buccal ceramic stratification) N.B.: crowns, bridges, and full-arch rehabilitations were included	Divides groups in follow up time instead of prosthetic restoration type.	Up to 5 years (average not specified)	Not specified (we assume 100%)	Not specified (we assume 100%)	1 case of chipping
Pozzi A. et al. (2021) [29]	Retrospective Clinical Study	98 patients	111 arches (96 zirconia connection, 15 titanium base) All frameworks were cutback and veneered.	24 complete ISZFDPs with a zirconia connection (12.9 ± 0.97 dental units, minimum 12, maximum 14), 72 partial prostheses with a zirconia connection (3.11 ± 1.12, minimum 2, maximum 7), 15 partial prostheses with a titanium base (3.62 ± 1.02, minimum 2, maximum 5).	Forty ISZFDPs had been in function for more than 10 years (36%), 38 for 5 to 9 years (34.2%), and 33 for 2 to 4 years (22.8%). The mean follow-up time was 7.2 ± 3.4 years.	98.2%	91.9%	No zirconia fractures 2 implants and 2 ISFCDPs failed due to chipping (13.5%) The 4 different types of veneering porcelains experienced the following chipping rates: ZI-CT Creation Willi Geller (0 out of 6; 0%), CZR (6 out of 77; 7.8%), IPS e.max Ceram (7 out of 26; 26.9%), and NobelRondo (2 out of 2; 100%). None of the ISZFDPs had to be remade because of esthetic reasons
Tirone F. et al. (2021) [30]	Retrospective Clinical Study	140 patients	180 arches in monolithic zirconia	Group 1: completely veneered zirconia IFCDP (21 ISFCDP) Group 2: zirconia IFCDP with veneering only on the buccal surface of all teeth (71 ISFCDP) Group 3: monolithic zirconia IFCDP veneered in the gingival portion only (41 ISFCDP)	Min. 12 months, max 87 months MEAN: 41.6 ± 21.2 months	Not specified	93.3%	2 prosthetic failures due to implant failures 8 framework fractures (5 type I, all maxillary, and 5 type II, all mandibular)

## Data Availability

Not applicable.
